# The Differences in Radiographic Vertebral Size in Dogs with Different Chest and Skull Types

**DOI:** 10.3390/ani14030470

**Published:** 2024-01-31

**Authors:** Amonsiri Phansangiemjit, Kamolphatra Kasemjiwat, Krit Patchanee, Yossapat Panninvong, Ana Sunisarud, Nan Choisunirachon, Chutimon Thanaboonnipat

**Affiliations:** 1 Faculty of Veterinary Science, Chulalongkorn University, Henri-Dunant Road, Pathumwan, Bangkok 10330, Thailand; amonsiri.phan@gmail.com (A.P.); jennykasem@gmail.com (K.K.); omgpcn@gmail.com (K.P.); yossapat.pnv@gmail.com (Y.P.); anapearanapear@gmail.com (A.S.); 2 Department of Veterinary Surgery, Faculty of Veterinary Science, Chulalongkorn University, Henri-Dunant Road, Pathumwan, Bangkok 10330, Thailand; nan.c@chula.ac.th

**Keywords:** canine, radiography, vertebral height, vertebral length, vertebral length to height ratio

## Abstract

**Simple Summary:**

Several proposed criteria for the comparative radiographic assessment of organs including heart, kidneys, small intestine, and large intestine, with vertebral measurement for objective comparison and normalization. However, considering the different body sizes and conformations of dogs, the proposed normal range for each comparative radiographic assessment might not be suitable for all dogs. The objective of this study was to elucidate the differences in vertebral length, vertebral height, and vertebral length/height ratio of the fourth thoracic vertebra (T4), the second lumbar vertebra (L2), the fifth lumbar vertebra (L5) and the seventh lumbar vertebra (L7) that are usually used as comparative parameters on radiographs in dogs with various body sizes, skull types, and thoracic conformations and to determine the relationships of these parameters with age and sex. Based on our findings, age, differences in body size, skull type, and thoracic conformation could affect the vertebral size in dogs.

**Abstract:**

The objective of this study was to elucidate the differences in vertebral length, vertebral height, and vertebral length/height ratio of the fourth thoracic vertebra (T4), the second lumbar vertebra (L2), the fifth lumbar vertebra (L5) and the seventh lumbar vertebra (L7) based on radiographs in dogs with various body sizes, skull types, and thoracic conformations and to determine the relationships of these parameters with age and sex. A total of 258 dogs were included in this study and classified by three criteria—BW (Criterion 1), skull type (Criterion 2), and thoracic conformation (Criterion 3). Age had weak negative correlations with vertebral length and height. Sex did not affect the vertebral size parameters. BW had strong positive correlations with vertebral length and height, but there was no influence of BW on vertebral length/height ratio. Regarding the different body sizes and conformations, large breeds had vertebrae with significantly greater length and height than small and medium breeds (*p* < 0.001). In Criterion 2, the vertebrae of the mesocephalic dogs had significantly greater length and height than those of the brachycephalic and dolichocephalic dogs (*p* < 0.05). In Criterion 3, both deep-chest and round-chest dogs had vertebrae with significantly greater length and height than the barrel-chest dogs (*p* < 0.0001). Only vertebral length/height ratios of T4 were not influenced by age, sex, BW, skull type, and thoracic conformation. Age, differences in body size, skull type, and thoracic conformation could affect the vertebral size in dogs. Therefore, using breed-specific vertebral lengths and/or heights is a better approach for comparative radiographic analysis with vertebral measurements.

## 1. Introduction

The vertebral column is the central axis of the skeleton in vertebrate animals including humans and dogs [1]. Canine vertebrae consist of five groups according to their location and morphology, namely cervical (C), thoracic (T), lumbar (L), sacral (S), and caudal (Cd) vertebrae [1,2].

In clinical practice, the vertebral column is generally used as the landmark on radiographs in evaluating organ size and diagnose abnormalities. Several proposed criteria for radiographic evaluations of the organ size in dogs, including heart, kidneys, small intestine, and large intestine, used vertebral length and height as the key parts for objective comparison and normalization [3]. The popular method for heart size evaluation, known as the vertebral heart score (VHS), is the method that compares the sum of cardiac dimensions to the number of the fourth thoracic vertebrae (T4) [4]. The first report of the VHS reference range in dogs was 9.2–10.2 vertebrae [4]. However, considering various breeds of dogs that have different body sizes and conformations [5] (Marin et al., 2007), a breed-specific VHS has been continuously investigated. Most breed-specific VHS values are higher than the original VHS proposed [4]. The range of VHS in Pugs and Beagles is 9.8–11.6 vertebrae and 9.9–10.7 vertebrae, respectively [6,7], indicating that interbreed variations in thoracic vertebrae cause variations in VHS values.

Apart from the VHS, radiographic renal size as well as small and large intestines are also determined by comparing them to the length of L, including second lumbar vertebrae (L2) [8], fifth lumbar vertebrae (L5) [9], and seventh lumbar vertebrae (L7) [10]. In previous studies, the Miniature Schnauzer had naturally short vertebrae compared to other breeds [4,11] and higher VHS and renal size/L2 ratios than the standard reference of normal dogs, resulting in false-diagnosed cardiomegaly [4] and renomegaly [11]. However, in dogs, information regarding vertebral length, height, and length/height ratio to explain to the real shape of vertebrae is limited.

Therefore, the objectives of this study were to elucidate the differences in vertebral length, height, and length/height ratio of T4, L2, L5, and L7 based on radiographs in dogs with various body sizes, skull types, and thoracic conformations. In addition, we determined the relationships of vertebral length, height, and length/height ratio based on radiographs with age, sex and bodyweight (BW).

## 2. Materials and Methods

### 2.1. Animals

This study was designed as a retrospective observational study. All information, including clinical demographic data, history, and all images, were allowed to be used by the committee of the Small Animal Hospital, Faculty of Veterinary Science, Chulalongkorn University (Approval number: 155/2564 on 1 June 2021. This study used the clinical data as well as thoracic and abdominal radiographs of dogs presented to the Diagnostic Imaging Unit, Small Animal Hospital, Faculty of Veterinary Science, Chulalongkorn University, between January 2017 and December 2020. The data were retrieved from the Hospital Information System (HIS) and Picture Archiving and Communication System (PACS). Clinical information including age, sex, gonadal status, breed, and BW was recorded. The inclusion criteria were good positioning and good covering of thoracic and/or abdominal radiographic images which covered from thoracic inlet to last rib for thoracic radiograph and covered from cupula of diaphragm to greater trochanter of femur for abdominal radiograph. The exclusion criteria were immature dogs that presented incomplete growth plate closure, dogs that had any abnormalities of vertebrae such as fractures, hemivertebrae, kyphosis, lordosis, scoliosis, and arthrosis.

All included dogs were classified into three main criteria based on their BW, skull type, and thoracic conformation. Criterion 1 was divided based on BW, as described by the American Kennel Club (AKC) standard [12], which were (1) small breed (BW less than 10 kg), (2) medium breed (BW between 10 and 24 kg), and (3) large breed (BW between 25 and 40 kg) [13,14]. Criterion 2 was divided based on skull types, which were (1) dolichocephalic, (2) mesocephalic, and (3) brachycephalic [15]. Criterion 3 was divided based on thoracic conformations, which were (1) deep-chest, (2) round-chest (or intermediate-chest), and (3) barrel-chest (or broad-chest) [16,17].

### 2.2. Experimental Procedures

#### Radiographic Measurement and Evaluation

**Figure 1:**
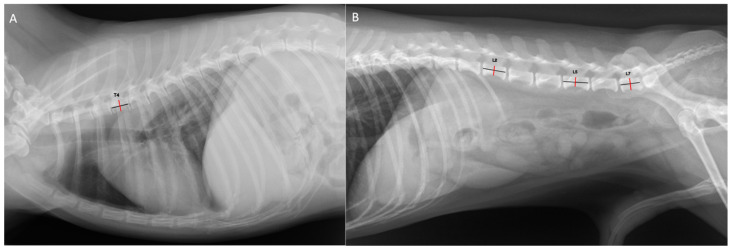
The vertebral length and height measurement methods on lateral radiographs. The vertebral length was measured from cranial ridge to caudal ridge of each vertebra (black line) and the vertebral height was measured from the narrowest part of each vertebra (red line). The vertebral length and height measurement of T4 (**A**). The vertebral length and height measurement of L2, L5 and L7 (**B**).

The right lateral projections of thoracic and abdominal radiographs of all included dogs were retrieved from the PACs. The included radiographs were obtained from digital radiograph (ETL^®^, GE healthcare, Chicago, IL, USA) with 65 kVp and 10 mAs. All images were collected as the digital information and communication file (DICOM) for measuring the vertebral length and height through the DICOM viewer software version 3.3.3 (Horos™, Horos Project, Annapolis, MD, USA). First, all radiographs that met the inclusion criteria were retrieved. Subsequently, the length and height of the fourth thoracic vertebra (T4), second lumbar vertebra (L2), fifth lumbar vertebra (L5), and seventh lumbar vertebra (L7) were measured and recorded. Vertebral length was measured from the cranial to the caudal ridge of each vertebra (the vertebral body), and vertebral height was measured from the narrowest part of each vertebra, as shown in Figure 1. The vertebral length/height ratios (length/height ratios) of T4, L2, L5, and L7 were then calculated. All the measurements were performed by well-trained veterinarians under supervision by radiologist specialist.

### 2.3. Statistical Analysis

All data were analyzed using statistical software (Prism 9, GraphPad^®^, San Diego, CA, USA). All data sets were analyzed by the Shapiro–Wilk test to test for data normalization. The descriptive data of clinical information are expressed as the mean ± standard deviation (SD) in the case of normal distribution and expressed as the median and range if the data were not normally distributed. Vertebral length, height, and length/height ratios were analyzed using one-way ANOVA or the Kruskal–Wallis test, followed by Tukey’s or Dunn’s test. All parameters were compared between sexes and gonadal statuses, using the unpaired T-test and the Mann–Whitney U test. The correlations and associations of each parameter with other factors, such as age and BW, were evaluated by using Pearson’s correlation coefficient and Spearman’s correlation coefficient. A *p*-value less than 0.05 indicated statistical significance.

## 3. Results

### 3.1. Clinical Demographic Data

A total of 258 dogs were included in this study. The breeds were Poodle (*n* = 44, 17.12%), Golden Retriever (*n* = 32, 12.45%), Pug (*n* = 26, 10.12%), Shih Tzu (*n* = 25, 9.73%), Siberian Husky (*n* = 22, 8.56%), French Bulldog (*n* = 19, 7.39%), Labrador Retriever (*n* = 19, 7.39%), Pomeranian (*n* = 16, 6.23%), Dachshund (*n* = 8, 3.11%), German Shepherd (*n* = 6, 2.33%), Beagle (*n* = 5, 1.95%), Pit Bull (*n* = 4, 1.56%), Yorkshire Terrier (*n* = 4, 1.56%), Alaskan Malamute (*n* = 3, 1.17%), Cocker Spaniel (*n* = 3, 1.17%), Pembroke Welsh Corgi (*n* = 3, 1.17%), Japanese Splitz (*n* = 3, 1.17%), American Pit Bull Terrier (*n* = 2, 0.78%), Akita (*n* = 2, 0.78%), Bulldog (*n* = 2, 0.78%), St. Bernard (*n* = 2, 0.78%), American Bully (*n* = 1, 0.40%), Bichon Frise (*n* = 1, 0.40%), Bull Terrier (*n* = 1, 0.40%), Dobermann (*n* = 1, 0.40%), Gordon Setter (*n* = 1, 0.40%), Peking (*n* = 1, 0.40%), and Shetland Sheepdog (*n* = 1, 0.40%). There were 155 male dogs (37 castrated and 118 intact dogs) and 102 female dogs (32 spayed and 70 intact dogs). All clinical demographic information of the included dogs is summarized in Table 1.

All dogs were reviewed and categorized into three main criteria. The clinical demographic information of dogs in each criterion is summarized in Table 2.

### 3.2. Radiographic Vertebral Parameters

We evaluated three parameters, namely vertebral length, vertebral height, and length/height ratios of T4, L2, L5, and L7 in each criterion.

**Figure 2:**
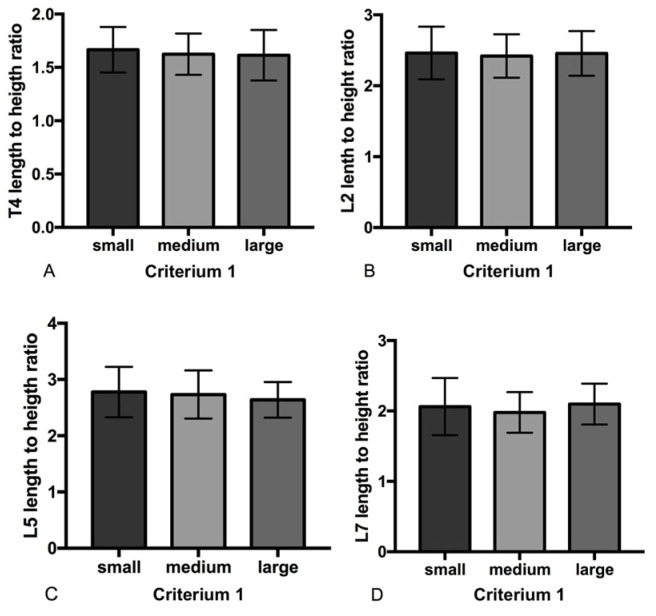
Comparing vertebral length/height ratios among size criteria (mean ± SD). (**A**) T4 length/height ratios, (**B**) L2 length/height ratios, (**C**) L5 length/height ratios and (**D**) L7 length/height ratios. Statistical differences among criteria were found using one-way ANOVA followed by Tukey’s test.

For Criterion 1, the results showed that the large-breed dogs had a significantly higher vertebral length and height of T4, L2, L5, and L7 than the small- and medium-breed dogs (*p* < 0.001). The L2, L5, and L7 lengths of medium-breed dogs were significantly higher than those of small-breed dogs (*p* < 0.0001, *p* = 0.002, and *p* < 0.0001, respectively). In contrast, the T4 lengths of medium-breed and small-breed dogs were not significantly different. The T4, L2, and L7 heights of medium-breed dogs were significantly higher than those of small-breed dogs (*p* = 0.002, *p* = 0.0345, and *p* = 0.0132, respectively), but there were no significant differences in L5 height between medium- and small-breed dogs. Interestingly, no significant difference in the length/height ratios among the size criteria was detected (Figure 2).

**Figure 3:**
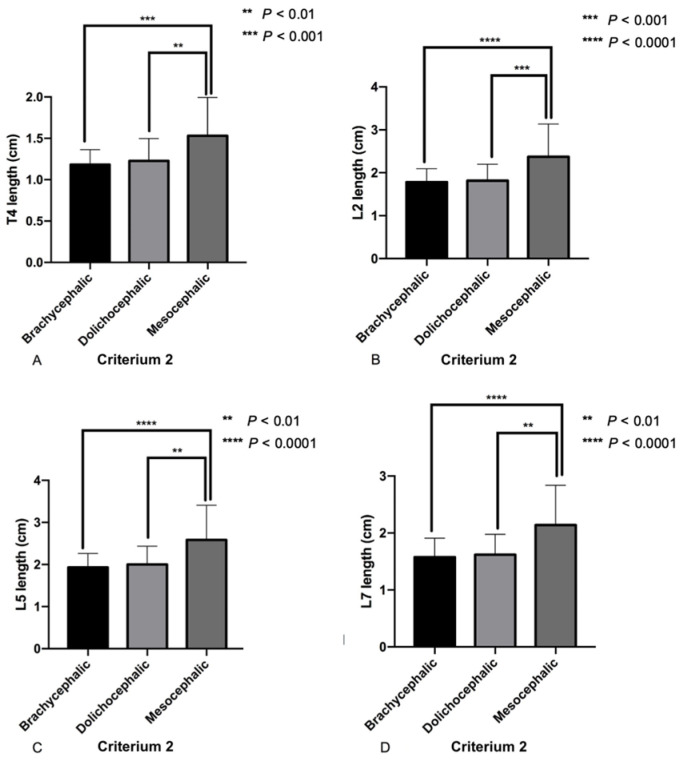
The vertebral length of mesocephalic dogs had significantly longer vertebrae of all four vertebrae than those of brachycephalic and dolichocephalic dogs (mean ± SD). (**A**) T4 length, (**B**) L2 length, (**C**) L5 length and (**D**) L7 length. Statistical differences among skull criteria were found using one-way ANOVA or the Kruskal–Wallis test followed by Tukey’s test.

For Criterion 2, the mesocephalic dogs had significantly longer vertebrae than the brachycephalic and dolichocephalic dogs (*p* < 0.001; Figure 3). No significant difference in vertebral length between brachycephalic and dolichocephalic dogs was detected. Regarding vertebral height, T4, L2, and L7 of the mesocephalic skull type were significantly higher than those of the brachycephalic and dolichocephalic dogs (*p* < 0.05, *p* < 0.05, and *p* < 0.001, respectively). In contrast, the L5 of mesocephalic dogs were significantly higher than those of dolichocephalic dogs (*p* < 0.001), but no significant difference between mesocephalic and brachycephalic dogs was detected. In the comparison of length/height ratios among skull type criteria, only T4 showed no significant difference among skull types.

**Figure 4:**
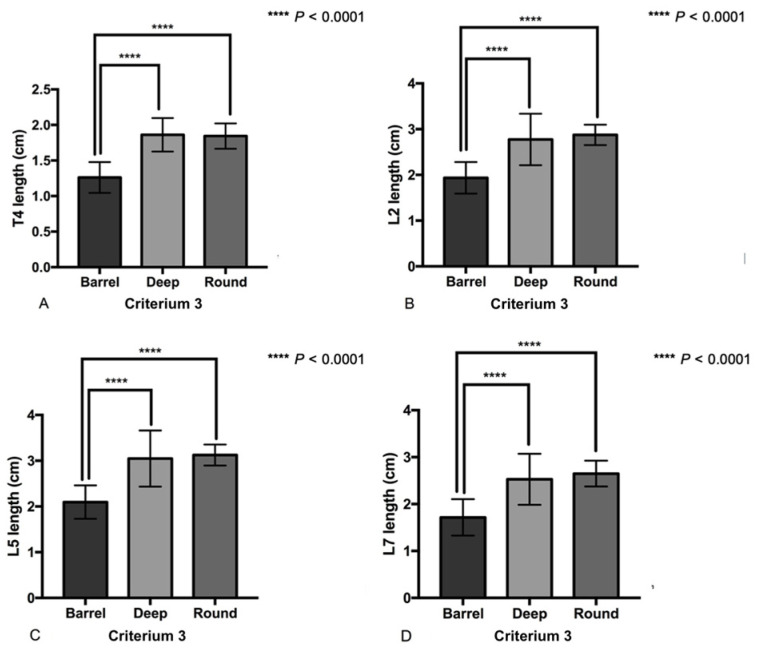
The vertebral length of both deep-chest and round-chest group had significantly longer vertebrae of all four vertebrae than that of barrel-chest dogs (mean ± SD). (**A**) T4 length, (**B**) L2 length, (**C**) L5 length and (**D**) L7 length. Statistical differences among thoracic conformation criteria were found using one-way ANOVA or the Kruskal–Wallis test followed by Tukey’s test.

For Criterion 3, both the deep-chest and the round-chest dogs had significantly longer vertebrae than the barrel-chest dogs (*p* < 0.0001; Figure 4). In contrast, no significant difference in vertebral length between the deep-chest and the round-chest dogs was detected. Similarly, the vertebral heights of the deep-chest and the round-chest dogs were significantly higher than that of the barrel-chest dogs (*p* < 0.05 and *p* < 0.0001, respectively). Interestingly, round-chest dogs had higher or taller vertebrae than deep-chest dogs (*p* < 0.05), except for T4, which showed no significant difference. However, the T4 height of the round-chest dog was greater than that of the deep-chest dogs. For the length/height ratios, deep-chest dogs had the highest values, followed by round-chest and barrel-chest dogs. No significant difference in the length/height ratio between round-chest and barrel-chest dogs was detected, and only T4 showed no significant difference among criteria.

### 3.3. Correlations of Vertebral Parameters with Age, Sex, and BW

**Figure 5:**
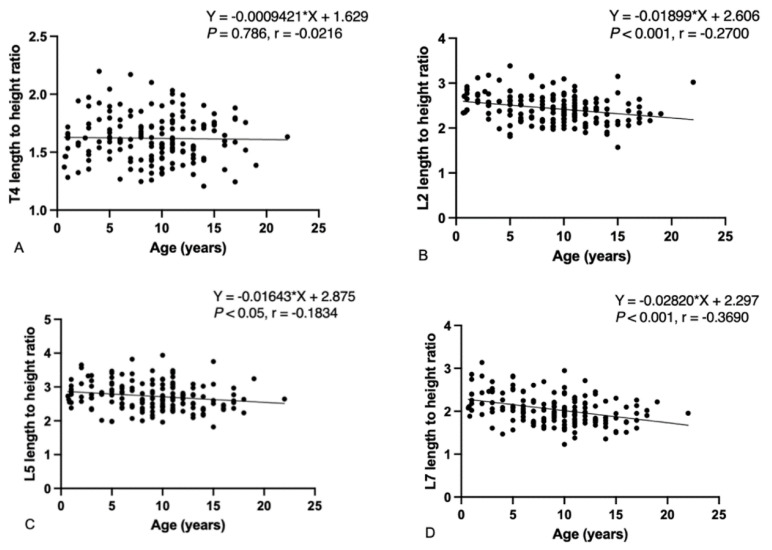
Correlations between the ages of all dogs and the vertebral length/height ratio. (**A**) T4 length/height ratio, (**B**) L2 length/height ratio, (**C**) L5 length/height ratio and (**D**) L7 length/height ratio. Correlations between parameters were made using Pearson’s correlation coefficient and Spearman’s correlation coefficient.

Age had non-significant negative weak correlations with vertebral length and height. However, age had significant negative weak correlations with the length/height ratios of L2, L5, and L7 (L2: r = −0.270; *p* < 0.001, L5: r = −0.183; *p* < 0.05; and L7: r = −0.369; *p* < 0.001). No significant correlation between age and T4 length/height ratios was detected (T4: r = −0.021; *p* = 0.786) (Figure 5). Male dogs had both greater vertebral lengths and heights than female dogs in all groups. No significant difference between gonadectomized and intact dogs in both males and females was detected. The overall BW showed significantly strong positive correlations with vertebral length (T4: r = 0.892; *p* < 0.001, L2: r = 0.901; *p* < 0.001, L5: r = 0.896; *p* < 0.001 and L7: r = 0.832; *p* < 0.001) and height (T4: r = 0.862; *p* < 0.001, L2: r = 0.873; *p* < 0.001, L5: r = 0.876; *p* < 0.001 and L7: r = 0.875; *p* < 0.001). In contrast, BW showed non-significantly weak negative correlations with length/height ratios (T4: r = −0.010; *p* = 0.153, L2: r = −0.028; *p* = 0.687, L5: r = −0.221; *p* = 0.140; and L7: r = −0.003; *p* = 0.961). The BW of skull type criteria showed significantly strong positive correlations with vertebral length (T4: r = 0.867; *p* < 0.001, L2: r = 0.887; *p* < 0.001, L5: r = 0.875; *p* < 0.001 and L7: r = 0.805; *p* < 0.001) and height (T4: r = 0.792; *p* < 0.001, L2: r = 0.812; *p* < 0.001, L5: r = 0.794; *p* < 0.001 and L7: r = 0.808; *p* < 0.001), while BW showed non-significantly weak negative correlations with length/height ratios (T4: r = −0.116; *p* = 0.188, L2: r = −0.028; *p* = 0.754, L5: r = −0.172; *p* = 0.06; and L7: r = −0.042; *p* = 0.632). In the same direction, the BW of dogs classified by thoracic conformation criteria showed significantly strong positive correlations with vertebral length (T4: r = 0.823; *p* < 0.001, L2: r = 0.826; *p* < 0.001, L5: r = 0.782; *p* < 0.001 and L7: r = 0.832; *p* < 0.001) and height (T4: r = 0.790; *p* < 0.001, L2: r = 0.808; *p* < 0.001, L5: r = 0.702; *p* < 0.001 and L7: r = 0.810; *p* < 0.001). In contrast, BW also showed non-significantly correlations with length/height ratios (T4: r = −0.332; *p* = 0.140, L2: r = −0.274; *p* = 0.229, L5: r = −0.206; *p* = 0.369; and L7: r = −0.130; *p* = 0.572).

## 4. Discussion

In this present study, the results revealed that age was negatively correlated with vertebral length and height. These findings correspond with those previously reported for humans and dogs in which degenerative changes in the vertebral column increase with age due to senile osteoporosis or other causes of an age-related decrease in bone density [18,19,20,21,22]. On comparison with humans, due to differences in body posture between humans and dogs, canine vertebral length would be compared to the human vertebral height and vice versa [22]. Additionally, only the length/height ratio of T4 was not affected by age.

Considering the effect of sex on vertebral parameters, our results showed that sex did not affect the vertebral length, height, and length/height ratio of T4, L2, L5, and L7. Our results are consistent with previous results reported in dogs showing that there is no significant difference in the T4 length and height between the sexes [23]. However, these results are in contrast to those found in humans [24], namely that males have an increased vertebral height compared to women [24]. As previously reported for humans [25], dogs [26], cat [27] and marine mammals [28], sexual dimorphism and sex hormones play significant roles in the growth and maintenance of the skeletal system, resulting in a larger body size of males compared to females [29]. In this study, although male vertebrae were on average 58% longer and 53% higher than females, this difference was not significant.

The overall BW regarding the body size of dogs positively correlated with the vertebral lengths and heights of T4, L2, L5, and L7, whereas no influence of BW on the vertebral length/height ratio was detected. This is in agreement with a previous study in humans that vertebral body height was positively correlated with human body length at all levels of the vertebrae and BW at levels L1 to L3 [22]. The reason why BW in humans has an influence at levels L1 to L3 might be the morphological differences between humans and dogs. It has been reported that, in English bulldog, front limbs bear 67.3% of the BW and hind limbs carry 32.6% [30]. These results are comparable to the distribution in other small, regular, and large breed dogs [31,32,33], whereas most of the weight in humans is carried by the lower limbs [34].

Regarding the radiographic vertebral parameters of the skull classification (Criterion 2), the mesocephalic dogs had a longer and higher vertebrae shape than the dolichocephalic and brachycephalic dogs, whereas no significant difference in vertebral length and height was observed between brachycephalic and dolichocephalic dogs. Interestingly, only the T4 length/height ratio showed a non-significant difference among skull types, whereas the length/height ratios of L2, L5, and L7 in mesocephalic dogs were significantly higher than those in brachycephalic dogs. These findings lead us to infer that the vertebrae of mesocephalic dogs are more rectangle in shape than those of dolichocephalic and brachycephalic dogs, whereas brachycephalic dogs have rather square vertebrae.

In the group of thoracic conformation (Criterion 3), deep-chest and round-chest dogs had a longer and higher vertebrae shape than barrel-chest dogs. Round-chest dogs had significantly higher vertebrae than deep-chest dogs, except for T4, whereas deep-chest dogs had the highest length/height ratios, followed by round-chest and barrel-chest ones. Based on our findings, deep-chest dogs have more rectangle vertebrae than round-chest and barrel-chest dogs. On the contrary, round-chest and barrel-chest dogs have similar vertebral shapes, with a rather square appearance.

Only the length/height ratios of T4 were not influenced by age, sex, BW, skull type, and thoracic conformation. The reason might be because on the radiographic images the best focusing of the image will be go to the center, which is the T4 location, while other vertebrae may not be suitably focused and cause inaccuracies in measurement.

The limitation of this study was the small sample size of included dogs in each categorized group. Therefore, further studies with a large number of samples should be considered. Another limitation was the broad inclusion criteria of each criterion that include various breeds of dogs in this study. However, the results of this study provide important information in which the radiographic vertebral size is different among dogs with different body sizes, skull types and chest conformations.

## 5. Conclusions

This study demonstrates that age and BW can influence the height and length of the vertebrae in dogs. The values decreased with age (after the growth plate closure) and increased with increasing BW. The length/height ratios were not affected by other parameters including age, sex, and BW. The radiographic vertebrae dimensions change with age, chest type and skull type. Therefore, using breed-specific vertebral lengths and/or heights is a better approach for comparative radiographic analysis with vertebral measurements.

## Figures and Tables

**Table 1:** Clinical demographic information of all included dogs in this study.

Clinical Information			Mean ± SD	Range
Age (years)	Overall		8.07 ± 4.49	0.67–22.00
	Male	Overall	7.99 ± 4.26	0.67–18.00
		Intact	7.88 ± 4.43	0.67–18.00
		Castrated	8.34 ± 3.71	1–15
	Female	Overall	8.20 ± 4.83	0.75–22.00
		Intact	7.26 ± 4.89	0.75–19.00
		Spayed	10.25 ± 4.05	4.00–22.00
BW (kg)	Overall		17.59 ± 13.48	1.30–80.25
	Male	Overall	18.60 ± 14.35	2.40–80.25
		Intact	18.25 ± 14.32	2.40–80.25
		Castrated	19.71 ± 14.58	2.60–55.00
	Female	Overall	16.08 ± 11.97	1.30–45.80
		Intact	15.73 ± 11.71	1.30–41.00
		Spayed	16.86 ± 12.69	1.75–45.80

**Table 2:** Clinical demographic information of dogs in each group.

Clinical Information	Criteria								
	Criterion 1			Criterion 2			Criterion 3		
	Small (*n* = 81)	Medium (*n* = 77)	Large (*n* = 98)	Dolichocephalic (*n*= 59)	Mesocephalic (*n* = 65)	Brachycephalic (*n*= 65)	Deep-Chest (*n* = 45)	Round-Chest (*n* = 34)	Barrel-Chest (*n* = 41)
*Male*	50	46	59	34	41	38	26	21	25
Intact	36	38	44	26	28	27	21	13	20
Castrated	14	8	15	8	13	11	5	8	5
*Female*	31	31	39	25	24	27	19	13	16
Intact	22	21	26	15	17	18	15	8	11
Castrated	9	10	13	10	7	9	4	5	5
*Age (years)*									
Mean ± SD	7.76 ± 4.37	9.15 ± 4.80	7.56 ± 4.21	7.53 ± 4.71	8.04 ± 4.10	7.53 ± 4.71	6.10 ± 3.93	8.85 ± 3.73	6.32 ± 4.46
Range	1.00–18.00	0.67–22.00	0.67–17.00	1.00–22.00	1.00–19.00	0.67–18.00	1.00–17.00	1.00–17.00	0.67–15.00
Median	7.00	10.00	8.00	10.00	8.50	8.00	6.00	9.00	5.00
*BW (kg)*									
Mean ± SD	7.52 ± 3.37	12.69 ± 3.86	30.79 ± 9.81	10.10 ± 8.07	24.40 ± 15.54	11.02 ± 6.33	26.17 ± 13.59	33.83 ± 9.73	14.21 ± 6.38
Range	1.75–9.70	10.30–23.00	28.80–40.00	2.40–37.00	1.30–80.25	3.20–34.00	6.70–80.25	9.40–55.00	5.40–34.00
Median	7.30	12.15	29.00	7.80	23.7	9.48	24.50	35.00	13.00

## Data Availability

Data are available on request due to privacy or ethical restrictions.

## References

[1] Budras K.D., McCarthy P.H., Horowitz A., Berg R. (2007). Vertebral Column and Thorax. Anatomy of the Dog.

[2] Kayalioglu G. (2009). The spinal cord. The Vertebral Column and Spinal Meninges.

[3] Lee R., Leowijuk C. (1982). Normal parameters in abdominal radiology of the dog and cat. J. Small Anim. Pract..

[4] Buchanan J.W., Bücheler J. (1995). Vertebral scale system to measure canine heart size in radiographs. J. Am. Vet. Med. Assoc..

[5] Marin L.M., Brown J., McBrien C., Baumwart R., Samii V.F., Couto C.G. (2007). Vertebral heart size in retired racing Greyhounds. Vet. Radiol. Ultrasound..

[6] Kraetschmer S., Ludwig K., Meneses F., Nolte I., Simon D. (2008). Vertebral heart scale in the beagle dog. J. Small Anim. Pract..

[7] Jepsen-Grant K., Pollard R., Johnson L. (2013). Vertebral heart scores in eight dog breeds. Vet. Radiol. Ultrasound..

[8] Finco D.R., Stiles N.S., Kneller S.K., Lewis R.E., Barrett R.B. (1971). Radiologic estimation of kidney size of the dog. J. Am. Vet. Med. Assoc..

[9] Graham J.P., Lord P.F., Harrison J.M. (1998). Quantitative estimation of intestinal dilation as a predictor of obstruction in the dog. J. Small Anim. Pract..

[10] Owens J.M., Miery D.N. (1999). Radiographic interpretation for the small animal clinician. Gastrointestinal System.

[11] Sohn J., Yun S., Lee J., Chang D., Choi M., Yoon J. (2017). Reestablishment of radiographic kidney size in Miniature Schnauzer dogs. J. Vet. Med. Sci..

[12] AKC 2017 “Breed Weight Chart”. https://www.akc.org/expert-advice/nutrition/breed-weight-chart/.

[13] Salt C., Morris P.J., German A.J., Wilson D., Lund E.M., Cole T.J., Butterwicket R.F. (2017). Growth standard charts for monitoring bodyweight in dogs of different sizes. PLoS ONE.

[14] Seo E., Choi J., Choi M., Yoon J. (2013). Computed tomographic evaluation of cervical vertebral canal and spinal cord morphometry in normal dogs. J. Vet. Sci..

[15] Stone H.R., McGreevy P.D., Starling M.J., Forkman B. (2016). Associations between Domestic-Dog Morphology and Behavior Scores in the Dog Mentality Assessment. PLoS ONE.

[16] Corcoran B.M. (1991). Static respiratory compliance in normal dogs. J. Small Anim. Pract..

[17] Asorey I., Pellegrini L., Canfrán S., Ortiz-Díez G., Aguado D. (2020). Factors affecting respiratory system compliance in anaesthetised mechanically ventilated healthy dogs: A retrospective study. J. Small Anim. Pract..

[18] Bray J.P., Burbidge H.M. (1998). The canine intervertebral disk. Part Two: Degenerative changes--nonchondrodystrophoid versus chondrodystrophoid disks. J. Am. Anim. Hosp. Assoc..

[19] Skowrońska-Jóźwiak E., Płudowski P., Karczmarewicz E., Lorenc R.S., Lewiński A. (2010). Effect of sex, age, and anthropometric parameters on the size and shape of vertebrae in densitometric morphometry: Results of the EPOLOS study. Pol. Arch. Intern. Med..

[20] Bergknut N., Egenvall A., Hagman R., Gustås P., Hazewinkel H.A., Meij B.P., Lagerstedt A.S. (2012). Incidence of intervertebral disk degeneration-related diseases and associated mortality rates in dogs. J. Am. Vet. Med. Assoc..

[21] Demontiero O., Vidal C., Duque G. (2012). Aging and bone loss: New insights for the clinician. Ther. Adv. Musculoskelet. Dis..

[22] Salamon N.M., Van Langenhove C., Verstraete K.L. (2017). Height of Lumbar Disc and Vertebral Body: What Is the Relation with Body Mass Index, Subcutaneous Fat Thickness, Body Weight, Length and Age. https://core.ac.uk/download/pdf/200225088.pdf.

[23] Tangpakornsak T., Saisawart P., Sutthigran S., Jaturunratsamee K., Tachampa K., Thanaboonnipat C., Choisunirachon N. (2023). Thoracic Vertebral Length-to-Height Ratio, a Promising Parameter to Predict the Vertebral Heart Score in Normal Welsh Corgi Pembroke Dogs. Vet. Sci..

[24] Gilsanz V., Boechat M.I., Gilsanz R., Loro M.L., Roe T.F., Goodman W.G. (1994). Gender differences in vertebral sizes in adults: Biomechanical implications. Radiology.

[25] Wooten S.V., Moestl S., Chilibeck P., Alvero Cruz J.R., Mittag U., Tank J., Tanaka H., Rittweger J., Hoffmann F. (2021). Age-and sex- differences in cardiac characteristics determined by echocardiography in masters athletes. Front. Physiol..

[26] Uehara T., Orito K., Fujii Y. (2009). CT-based anatomical features of large airway and heart volume in dogs of different body size. Vet. J..

[27] Wanglerm A., Samachikthummakun C., Kitnitchee Z., Sirinitikorn T., Junnong S., Sutthigran S., Choisunirachon N., Thanaboonnipat C. (2023). Computed tomographic evaluation of heart size in clinically healthy cats. Open Vet. J..

[28] Ralls K., Mesnick S., Perrin W.F., Wuersig B., Thewissen J.G.M. (2009). Sexual dimorphism. Encyclopedia of Marine Mammals.

[29] Keutmann E.H. (1951). Sex Hormones and Growth. Med. Clin. N. Am..

[30] Mölsä S.H., Hyytiäinen H.K., Morelius K.M., Palmu M.K., Pesonen T.S., Lappalainen A.K. (2020). Radiographic findings have an association with weight bearing and locomotion in English bulldogs. Acta Vet. Scand..

[31] Levine D., Marcellin-Little D.J., Millis D.L., Tragauer V., Osborne J.A. (2010). Effects of partial immersion in water on vertical ground reaction forces and weight distribution in dogs. Am. J. Vet. Res..

[32] Hyytiäinen H.K., Mölsä S.H., Junnila J.T., Laitinen-Vapaavuori O.M., Hielm-Björkman A.K. (2012). Use of bathroom scales in measuring asymmetry of hindlimb static weight bearing in dogs with osteoarthritis. Vet. Compd. Orthop. Traumatol..

[33] Linder J.E., Thomovsky S., Bowditch J., Lind M., Kazmierczak K.A., Breur G.L., Lewis M.J. (2021). Development of a simple method to measure static body weight distribution in neurologically and orthopedically normal mature small breed dogs. BMC Vet. Res..

[34] McNeil C.J., Raymer G.H., Doherty T.J., Marsh G.D., Rice C.L. (2009). Geometry of a weight-bearing and non-weight-bearing bone in the legs of young, old, and very old men. Calcif. Tissue Int..

